# The challenges of severe acute respiratory syndrome coronavirus 2 (SARS-CoV-2) testing in low-middle income countries and possible cost-effective measures in resource-limited settings

**DOI:** 10.1186/s12992-022-00796-7

**Published:** 2022-01-22

**Authors:** Zamathombeni Duma, Anil A. Chuturgoon, Veron Ramsuran, Vinodh Edward, Pragalathan Naidoo, Miranda N. Mpaka-Mbatha, Khethiwe N. Bhengu, Nomzamo Nembe, Roxanne Pillay, Ravesh Singh, Zilungile L. Mkhize-Kwitshana

**Affiliations:** 1 Department of Medical Microbiology, School of Laboratory Medicine & Medical Sciences, Howard College, University of KwaZulu-Natal, Glenwood, Durban, 4041 South Africa; 2 Division of Research Capacity Development, South African Medical Research Council (SAMRC), Tygerberg, Cape Town, 7505 South Africa; 3 Department of Medical Biochemistry, School of Laboratory Medicine & Medical Sciences, Howard College, University of KwaZulu-Natal, Glenwood, Durban, 4041 South Africa; 4 The Aurum Institute, Parktown, Johannesburg 2194 South Africa; 5 Department of Biomedical Sciences, Mangosuthu University of Technology, Umlazi, Durban, 4031 South Africa

**Keywords:** SARS-CoV-2, Low-middle-income countries, Diagnostic testing challenges, Cost-effective strategies, Resource-limited settings

## Abstract

Diagnostic testing for the Severe Acute Respiratory Syndrome Coronavirus 2 (SARS-CoV-2) infection remains a challenge around the world, especially in low-middle-income countries (LMICs) with poor socio-economic backgrounds. From the beginning of the pandemic in December 2019 to August 2021, a total of approximately 3.4 billion tests were performed globally. The majority of these tests were restricted to high income countries. Reagents for diagnostic testing became a premium, LMICs either cannot afford or find manufacturers unwilling to supply them with expensive analytical reagents and equipment. From March to December 2020 obtaining testing kits for SARS-CoV-2 testing was a challenge. As the number of SARS-CoV-2 infection cases increases globally, large-scale testing still remains a challenge in LMICs**.** The aim of this review paper is to compare the total number and frequencies of SARS-CoV-2 testing in LMICs and high-income countries (HICs) using publicly available data from Worldometer COVID-19, as well as discussing possible interventions and cost-effective measures to increase testing capability in LMICs. In summary, HICs conducted more SARS-CoV-2 testing (USA: 192%, Australia: 146%, Switzerland: 124% and Canada: 113%) compared to middle-income countries (MICs) (Vietnam: 43%, South Africa: 29%, Brazil: 27% and Venezuela: 12%) and low-income countries (LICs) (Bangladesh: 6%, Uganda: 4% and Nigeria: 1%). Some of the cost-effective solutions to counteract the aforementioned problems includes using saliva instead of oropharyngeal or nasopharyngeal swabs, sample pooling, and testing high-priority groups to increase the number of mass testing in LMICs.

## Background

### Overview of SARS-CoV-2

In December 2019, the World Health Organization (WHO) reported several pneumonia cases in Wuhan, China [1]. Severe Acute Respiratory Syndrome Coronavirus 2 (SARS-CoV-2) was confirmed as the cause of Coronavirus disease 2019 (COVID-19) [2]. Severe Acute Respiratory Syndrome Coronavirus 2 is a positive-stranded ribonucleic acid (RNA) virus that primarily infects the upper respiratory tract and is associated with a wide range of complications, including lymphopenia, dyspnea, acute respiratory distress syndrome, pneumonia and acute cardiac arrest [3].

The virus contains four structural proteins namely the spike (S), membrane (M), envelope (E), and nucleocapsid (N) proteins [4]. SARS-CoV-2 entrance into the host cells is mediated by the spike protein. The receptor-binding domain that binds to the peptidase domain of angiotensin-converting enzyme 2 (ACE2) is located at the S1 subunit of the S protein [4, 5]. The M protein is the virus’s most abundant structural protein [4, 6] because it interacts with all of the other major coronaviral structural proteins, and it is assumed to be the central coordinator of coronavirus assembly [4]. The E protein is a minor component of the membrane. During the replication cycle, the E protein is widely expressed inside the infected cell, but only a small portion of it is integrated into the virion membrane [4, 7]. The nucleocapsid is made up of the viral RNA genome, and the N protein is the only protein that binds to it. The N protein is largely involved in viral genome activities, and also plays a role in other aspects of the viral replication cycle as well as the host cellular response to viral infection [4, 8].

Countries worldwide have been struggling to contain the highly contagious and rapidly mutating SARS-CoV-2 for more than a year [9]. As of 30 September 2021, the virus has spread to over a hundred countries, and about 222 million coronavirus cases had been confirmed worldwide, resulting in over 4,6 million deaths [10]. The WHO has declared COVID-19 a Public Health Emergency of International Concern [11, 12].

### Transmission of SARS-CoV-2 and incubation period

The main mode of transmission of SARS-CoV-2 is by person-person contact [13]. The virus is spread between people through minute aerosol droplets created by sneezing, coughing and talking during close contact. Another way a person can become infected with the virus is by surface transmission [14]. This is because the virus can live on surfaces for up to 96 h [13]. The virus is more likely to transmit through people who display symptoms early in the disease; this is known as symptomatic transmission [14]. In addition, there is a high chance of passing the virus without showing any signs and symptoms and this is known as an asymptomatic spread [15].

The virus transmission channel, the amount of virus that enters the host, and the interaction between the virus and the host immune system are all factors that influence the incubation period [16, 17]. According to the WHO and the Centers for Disease Control and Prevention (CDC), the incubation period for SARS-CoV-2 infection is estimated to be 1–14 days, with an average incubation period of around 5–6 days [17, 18]. According to a study conducted in Wuhan, China (January 2020 – February 2020), roughly 97.5% of SARS-CoV-2 infected patients exhibited clinical symptoms after 11.5 days, and the remaining 2.5% in 2.2 days [19]. To note, the incubation period varies from person to person [19]. One of the most serious concerns is that SARS-CoV-2 variants have evolved in huge numbers, causing transmission alterations [20]. However, there is limited information about the incubation duration for specific variants [20]. Therefore, more studies are needed to determine whether novel SARS-CoV-2 mutations affect the incubation period. Furthermore, that is the reason why the WHO still emphasizes the recommended quarantine period of 14 days [21].

### Safety measures for SARS-CoV-2 infection in LMICs

According to WHO (2020), the most recommended preventive measures for SARS-CoV-2 infection include social distancing, hand hygiene, using face masks and coughing in the elbow [22]. Implementing the recommended preventive measures in LMICs is a challenge due to unfavorable conditions such as overcrowding in the household, inadequate ventilation in dwellings, ambient and indoor air pollution, lack of clean water supply, refugee settings, the number of persons living on the streets, and poor sanitation [23]. Sanitation is a crucial issue in LMICs because a large number of people, particularly in rural and peri-urban regions, still rely on surface and groundwater sources for their daily water needs [24]. Pit toilets and groundwater are widely used in LMICs, while open defecation near surface water has also been reported as well [25]. The untreated effluent is dumped into the environment, potentially contaminating groundwater and surface resources [26]. As a result, this might partially contribute to the risk of SARS-CoV-2 transmission. Also, to be noted, a few studies reported detecting SARS-CoV-2 in wastewater which has epidemiologic potential and can be used as a backup technique to monitor viral tracking and circulation in places with limited SARS-CoV-2 testing capacity or highly populated regions where door-to-door tracing is difficult. However, in order to improve sensitivity, special attention must be paid to virus concentration and detection assays [27].

### The consequences of lockdown restrictions in LMICs

As a way to curb the spread of the rapidly mutating SARS-CoV-2, countries worldwide enforced strict lockdown restrictions [28]. By April 2020, more than 90 countries were in some form of lockdown. Stay-at-home orders, quarantine, isolation, social distancing, curfews, school and company closures, and travel restrictions are all part of the lockdown regulations [29]. The WHO proposed response to the SARS-CoV-2 outbreak involves personal hygiene, effective contact tracing, and isolation when an individual is infected, to strike a balance between lockdown restrictions and normalcy [30]. If implemented in a timely and comprehensive manner, lockdown can be an effective infection control and prevention mechanism, reducing the risk of virus transmission from person to person and population spread while buying enough time to scale up preventative measures, diagnostic tests, and treatment capability [31]. While the rigorous restrictions associated with lockdowns are effective, they come at a cost: they impose significant social and economic constraints on individuals and groups, particularly in LMICs [31].

Workers in the informal economy are affected the most by the lockdown because they lack social security and access to adequate health care, as well as having lost access to productive assets [32]. Hence, without the means to earn an income during lockdowns, many are unable to feed themselves and their families [33]. Due to border closures, trade barriers, and other restrictions, farmers are unable to access markets, causing a disruption in domestic and international food supply chains as well as limiting access to balanced, healthy, and diverse meals [34]. Therefore, millions of women, children and men’s food security have been compromised as a result of breadwinners losing their jobs due to the lockdown in low-income countries, with vulnerable communities such as small-scale farmers and indigenous people being the hardest hit [34].

Attempting to strengthen the economy of several LMICs, various governments have opted to ease lockdown restrictions. Therefore, lockdown restrictions in various countries were relaxed at various periods [35]. Currently, governments throughout the world are struggling to figure out whether and how to relax restrictions while balancing numerous health, social, and economic issues. Premature lifting of lockdown restrictions by allowing businesses to operate, opening schools and higher education institutions, and allowing traveling are among the key factors contributing to the resurgence of SARS-CoV-2 waves [36]. Hence, LMICs should try to learn from previous waves of SARS-CoV-2 infection and try to avoid being caught off guard by more waves of SARS-CoV-2 infection in the future [36]. This means it is crucial to develop methods that are cheaper, simple, and have a quick diagnostic turnaround time to avoid the medical laboratory staff becoming overwhelmed during the future waves.

### Challenges of SARS-CoV-2 testing in LMICs

Mass testing is one of the most significant aspects of lowering the SARS-CoV-2 infection rate through early detection of cases for treatment and subsequent cautionary measures such as isolation to prevent death and further virus transmission, respectively [37]. However, identification and monitoring of the SARS-CoV-2 infection cases have been the greatest challenge in the LMICs [37]. In LMICs, SARS-CoV-2 infection testing is problematic due to financial constraints and other factors [38]. These countries have no domestic capacity to manufacture nasopharyngeal swabs, analytical reagents and COVID 19 kits for SARS-CoV-2 testing [39]. With an increase in the number of SARS-CoV-2 infection cases, mass testing becomes disrupted due to a shortage of nasopharyngeal swabs, analytical reagents and COVID 19 kits. It is because buying all of the materials needed to test for SARS-CoV-2 infection is excessively expensive [40]. Furthermore, the cost of Personal Protective Equipment (PPE) has increased since the SARS-CoV-2 outbreak started, with LMICs bearing the brunt of the burden. The prices of surgical masks have increased sixfold, N95 breathing masks have tripled, and gowns have doubled. The problem is supply delivery could take months, and market manipulation is common, with inventories being sold to the highest bidder. This is concerning since healthcare workers rely on personal protective equipment to safeguard themselves and their patients from SARS-CoV-2 infections and the spread of infections. Therefore, doctors, nurses, and other frontline workers in LMICs are severely underequipped to care for SARS-CoV-2 patients because of limited access to equipment including gloves, medical masks, respirators, goggles, face shields, gowns, and aprons [41]. In addition, there are fewer laboratory staff trained for SARS-CoV-2 testing in LMICs. As the number of infection cases increases the laboratory staff becomes overwhelmed, and as a result, diagnostic turnaround time and transmission rates will be increased [40, 41].

Obtaining the best effective vaccine program and uneven access to vaccine programs are two other important concerns in LMICs. Vaccine distribution in the world remains highly unequal, with a majority of the existing supply going to high-income countries (HICs) [42]. Hence, it will take months to years for the COVID-19 vaccine to have an impact against the SARS-CoV-2 in LMICs. As a result, this is concerning because the vaccine program was supposed to be the way out of this crisis [43]. In 2021, millions of people in LMICs would be denied access to the COVID-19 vaccine due to wide disparities in COVID-19 vaccine access between HICs and LMICs [43]. As a consequence, the outbreak may be prolonged, increasing the risk of additional mutation and reducing the efficacy of current vaccines. Therefore, LMICs need to come up with innovative approaches to fight this contagious virus [20].

The major concern is how will LMICs deal with the SARS-CoV-2 pandemic? As a result, using publicly available data from Worldometer COVID-19 [10], this review paper will compare the total number and frequencies of SARS-CoV-2 testing in LMICs and HICs, as well as discussing possible interventions and cost-effective measures to increase testing capability in LMICs.

### Literature review and data sourcing

**Fig. 1:**
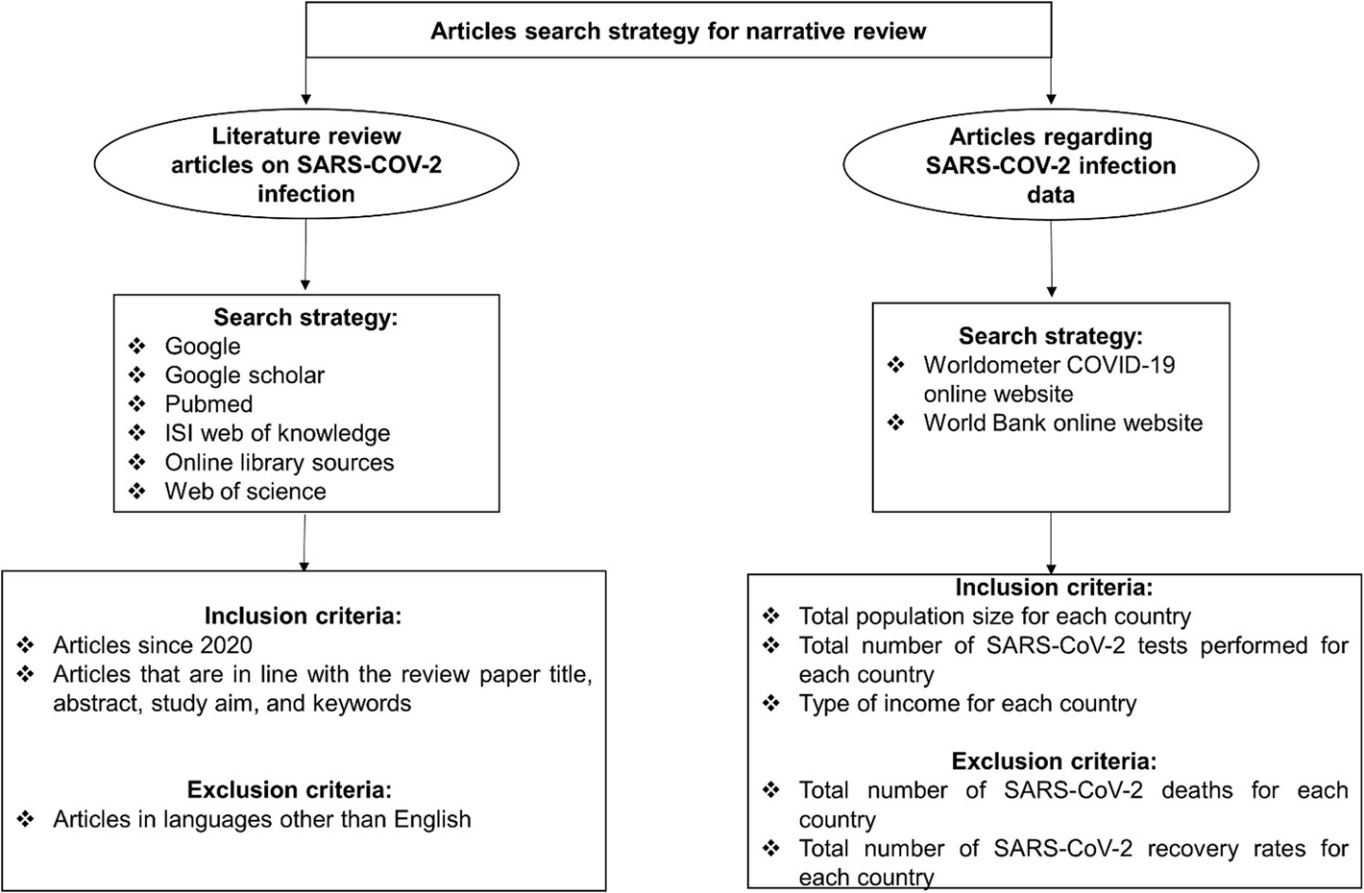
Prisma flow chart for SARS-CoV-2 literature articles and data search strategies

Article search strategies, inclusion and exclusion criteria and data sourcing for the study is presented in the Prisma flow diagram (Fig. 1).

## Results and overall findings

**Fig. 2:**
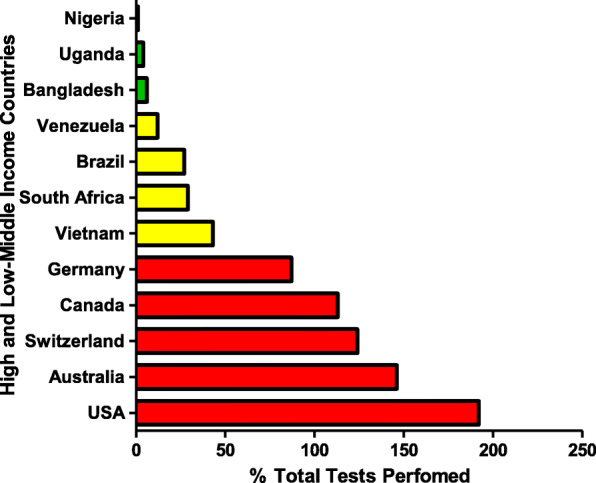
The number of SARS-CoV-2 tests (%) performed in high-income and middle-low-income countries and these samples represents counties of each income group. High-income countries are highlighted in red, middle-income countries are highlighted in yellow, and low-income countries are highlighted in green [10]. High-income countries (USA, Switzerland, Australia, Canada, Germany). Middle-income countries (Venezuela, Vietnam, South Africa, Brazil). Low-income countries (Bangladesh, Uganda, Nigeria)

**Fig. 3:**
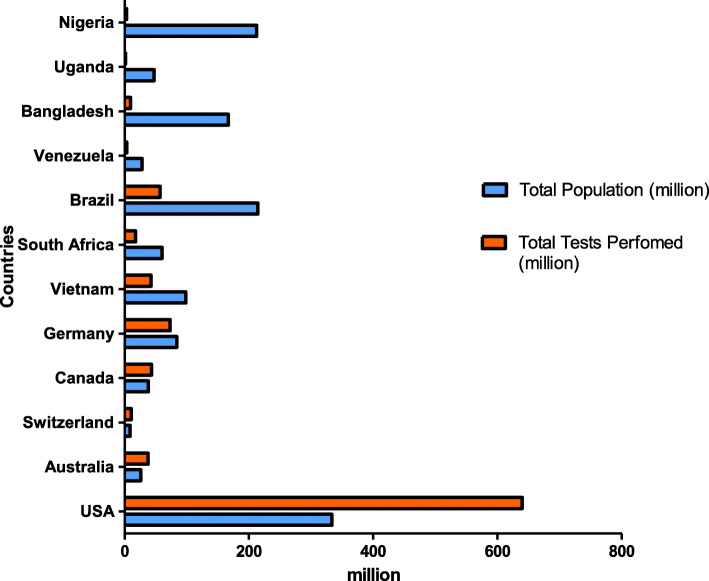
Comparison between the total population size (million) and the total number of SARS-CoV-2 tests performed (million) in each country [10]. High-income countries (USA, Switzerland, Australia, Canada, Germany). Middle-income countries (Venezuela, Vietnam, South Africa, Brazil). Low-income countries (Bangladesh, Uganda, Nigeria)

Table 1, Figs. 2 and 3 provide a comparison of the total number and frequencies of SARS-CoV-2 testing in each income group (low, middle, and high) and continent. The data in Table 1, Figs. 2 and 3 shows that high-income countries have undertaken 10 times more SARS-CoV-2 testing compared to LMICs [10]. More than 100% of the population in HIC (USA: 192%, Australia: 146%, Switzerland: 124% and Canada: 113%) has been tested for SARS-CoV-2, whereas only 27,5% of the population in middle-income countries (MIC) (Vietnam: 43%, South Africa: 29%, Brazil: 27% and Venezuela: 12%) and approximately 3% of the population in low-income countries (LIC) (Bangladesh: 6%, Uganda: 4% and Nigeria: 1%) has been tested.

**Table 1:** Comparison between the total number of tests (in million) performed and the total population (in million) in high-income countries and LMICs

Continent	Countries	Income	Total Tests Performed (million)	Total Population (million)	Percentage of Tests performed (%)
North America	USA	High	639,832,856	333,416,037	192^1^
Oceania	Australia	High	37,832,547	25,854,460	146^1^
Europe	Switzerland	High	10,796,404	8,733,303	124^1^
North America	Canada	High	43,215,201	38,153,447	113^1^
Europe	Germany	High	73,348,901	84,117,156	75
Asia	Vietnam	Middle	42,517,091	98,427,082	43
Africa	South Africa	Middle	17,649,727	60,237,549	29
South America	Brazil	Middle	57,282,520	214,437,809	27
South America	Venezuela	Middle	3,359,014	28,335,663	12
Asia	Bangladesh	Low	9,704,722	166,728,314	6
Africa	Uganda	Low	1,680,863	47,529,564	4
Africa	Nigeria	Low	2,997,060	212,473,029	1

Data were retrieved from the Worldometer Covid 19 on 30 September 2021 [10]. % Tests performed = (Total Tests/Total Population) * 100). The data on various types of income for each country was obtained from the World Bank online site [44]. ^(1)^ Excessive SARS-CoV-2 testing is indicated by percentage values above 100 (SARS-CoV-2 tests performed more than the actual population)

The possible reasons for under-testing for SARS-CoV-2 infection in LMICs are probably many people are unable to afford SARS-CoV-2 testing due to financial restrictions, unstable health systems and reliance on global supply chains. As a result, many positive cases are simply missed, putting LMICs at higher risk of spreading the virus [31]. The pandemic will easily shatter the poor health system and overburden hospitals and clinical services if effective prevention is not implemented [45].

### Possible cost-effective strategies to increase testing capability in LMICs

The management of SARS-CoV-2 infection cases entails early detection of the virus and prompt isolation as a result, which will aid in the prevention and control of virus spread [46]. Using cost-effective approaches such as saliva instead of oropharyngeal or nasopharyngeal swabs, sample pooling, testing high-priority groups and using antigen rapid tests can help to increase the number of mass testing in LMICs.

### Saliva sample for SARS-CoV-2 infection testing

The recommended sample type for SARS-CoV-2 detection is nasopharyngeal and/ or oropharyngeal swabs [47]. The problem with nasopharyngeal and oropharyngeal swabs is that it makes patients uncomfortable, forcing them to cough during sample collection and exposing health care workers to the high risk of infection. Furthermore, nasopharyngeal and oropharyngeal swabs are expensive because the sample has to be collected by trained health care personnel wearing PPE [48]. Therefore, the nasopharyngeal and oropharyngeal swab is not an ideal sample to utilize for monitoring SARS-CoV-2 load. Therefore, saliva could be used as an alternative sample for SARS-CoV-2 testing and viral load monitoring, due to its numerous advantages [49].

Saliva is a transparent biofluid generated by the salivary glands that clean and protects the oral cavity, has antibacterial properties, and aids in food digestion [50]. Angiotensin-converting enzyme 2 (ACE-2) has been identified as the principal host cell receptor of SARS-CoV-2, and it is thought to play a key role in the virus’s entry into the cell and subsequent infection. The ACE-2 receptor is highly expressed in the salivary gland and oral mucosa, [51] ACE2-positive cells in the salivary glands are likely to be SARS-CoV-2 target cells [52]. Furthermore, the presence of SARS-CoV-2 in saliva could be due to the mixing of upper and lower respiratory tract fluid that conveys the virus to the saliva. These findings imply that the salivary gland and oral mucosa could be a high-risk source site for SARS-CoV-2 infection [52]. Hence, this is what makes saliva a suitable specimen for testing SARS-CoV-2 infection.

Saliva could be utilized as a diagnostic sample for detecting SARS-CoV-2 and monitoring viral load [53]. Patients collect their own samples, which has several benefits, including the elimination of health care staff and the elimination of PPE for sample collection. The time, cost, and risk of viral transmission associated with sample collection are reduced, allowing for increased SARS-CoV-2 mass testing [53, 54]. Furthermore, saliva can be utilized efficiently in large organizations such as universities since PPE is not required, and this could help to lower the danger of viral transmission. Hence, the addition of saliva testing for SARS-Cov-2 infection will allow universities to test thousands of students and staff, with the aim that the results will aid in keeping campuses safe. As a result, saliva testing, in addition to wearing a face mask and maintaining social distance, is an innovative option [55]. However, less attention has been given to its potential usefulness in testing and monitoring for SARS-CoV-2 infection [54].

### Sample pooling

The gold standard for diagnosing SARS-CoV-2 infection is reverse-transcription polymerase chain reaction (RT–PCR), a molecular method [56]. Real-time PCR is precise, but it is expensive to test each individual regularly [57]. Therefore, high prices limit affordability for many people, particularly in LMICs. The cost savings can be achieved by pooling samples [57, 58].

The principle of sample pooling allows multiple samples to be mixed and tested as a single sample [59]. When using a pooling method and the pooled test result is negative, each batch component is treated as if it were analyzed separately. Individual testing is required only when the pool test results are positive [59]. Sample pooling testing should be recommended for asymptomatic suspected cases, excluding those who are symptomatic [60]. This method is advantageous because it is cost-effective and allows for increased mass testing for SARS-CoV-2 without compromising testing accuracy or wasting consumables such as analytical reagents and extraction kits [61]. As a consequence, this technique improves testing efficiency by reducing the backlog of SARS-CoV-2 pending tests while also enhancing diagnostic turnaround time, which is one of the most important elements in managing and controlling the SARS-CoV-2 outbreak [62]. The pooling technique will be extremely advantageous in a laboratory with limited resources because this type of testing is more feasible and less expensive for mass screening in a large community [63].

### Prioritized groups for testing of SARS-CoV-2 infection

It is critical to have a quick and accurate strategy for detecting and controlling SARS-CoV-2 outbreaks in communities and hospitals in LMICs [64]. In LMICs, prioritizing certain individuals for testing of SARS-CoV-2 infection should be considered. This testing strategy will help to accommodate the countries with limited resources by prioritizing individuals according to their categories of urgent clinical need while trying to reduce a backlog of pending testing [65]. Testing becomes the highest priority when it contributes to improving clinical outcomes and decreasing the transmission rate of the virus [66].

When prioritizing groups, the most important factors to consider are the size of each group, the number of tests needed, and the number of tests available. Hence, the most critical groups should be tested first. As testing becomes more generally available, it should be gradually spread to other groups based on their priorities. Additionally, those who tested positive for SARS-CoV-2 infection will need to undergo further testing [65].

A list of priority groups for SARS-CoV-2 testing in the private and public sectors is as follows: (i) Symptomatic patients, regardless of their age or underlying health issues, should be given the highest priority to reduce the risk of nosocomial transmission and protect health care staff and the general public. (ii) People who had contact with people who had tested positive for SARS-CoV-2 infection, whether asymptomatic or symptomatic, in order to quickly identify patients at high risk of complications and ensuring that the required precautions are taken. (iii) SARS-CoV-2 testing should also be prioritized for healthcare workers, frontline responders, essential critical infrastructure workers, miners, travelers, people going for surgery, testing pregnant women who are admitted at the labor ward and post-mortem testing, regardless of whether they are asymptomatic, to prevent a possible spread in the community and at work. (iv) If resources are available, testing for non-essential workers may be permitted [67, 68]. The most important thing to note is that healthy people who have not been tested should continue to practice social distance and wearing masks as recommended by their local and state health authorities [69, 70].

### Antigen rapid test as a screening test for SARS-CoV-2 infection in LMIC

As the world continues to wrestle with SARS-CoV-2 infections, the number of cases in LMIC are increasing, causing national economies to lock down and putting further strain on already struggling economies [71]. As a result, the antigen rapid test can be used as alternative strategy for SARS-CoV-2 infection in LMIC. Antigen rapid tests have the advantage of providing results in 15–30 min instead of in hours or days, allowing mass testing to be increased, especially in LMICs with limited laboratory facilities or qualified health professionals to do molecular (PCR) tests [72]. The antigen rapid test allows healthcare workers to quickly identify individuals who are infected with SARS-CoV-2, so they be isolated and treated while their contacts are tracked to prevent the virus from spreading to their families and communities. In this case of a SARS-CoV-2 outbreak, where the test turnaround time is crucial, antigen rapid tests play an important role in delivering early results [73].

While governments are increasingly relying on less expensive antigen rapid tests to increase SARS CoV-2 infection testing coverage, however, the test may have low sensitivity [74]. It’s critical to confirm an antigen test result with a PCR test, especially if the result of the antigen rapid test contradicts the clinical setting. Therefore, PCR tests remain the gold standard, and their value remains high [73, 75]. To be noted antigen rapid tests are typically used on symptomatic individuals since they perform best in symptomatic individuals and within a particular number of days of symptom onset [74]. By adopting this alternative strategy for mass testing of SARS-CoV-2 infection, LMICs can spend less money on diagnostics and more money on essential medical equipment for hospitals treating SARS-CoV-2 infected patients, resulting in more lives saved [72].

## Conclusion and implication for future research

**Fig. 4:**
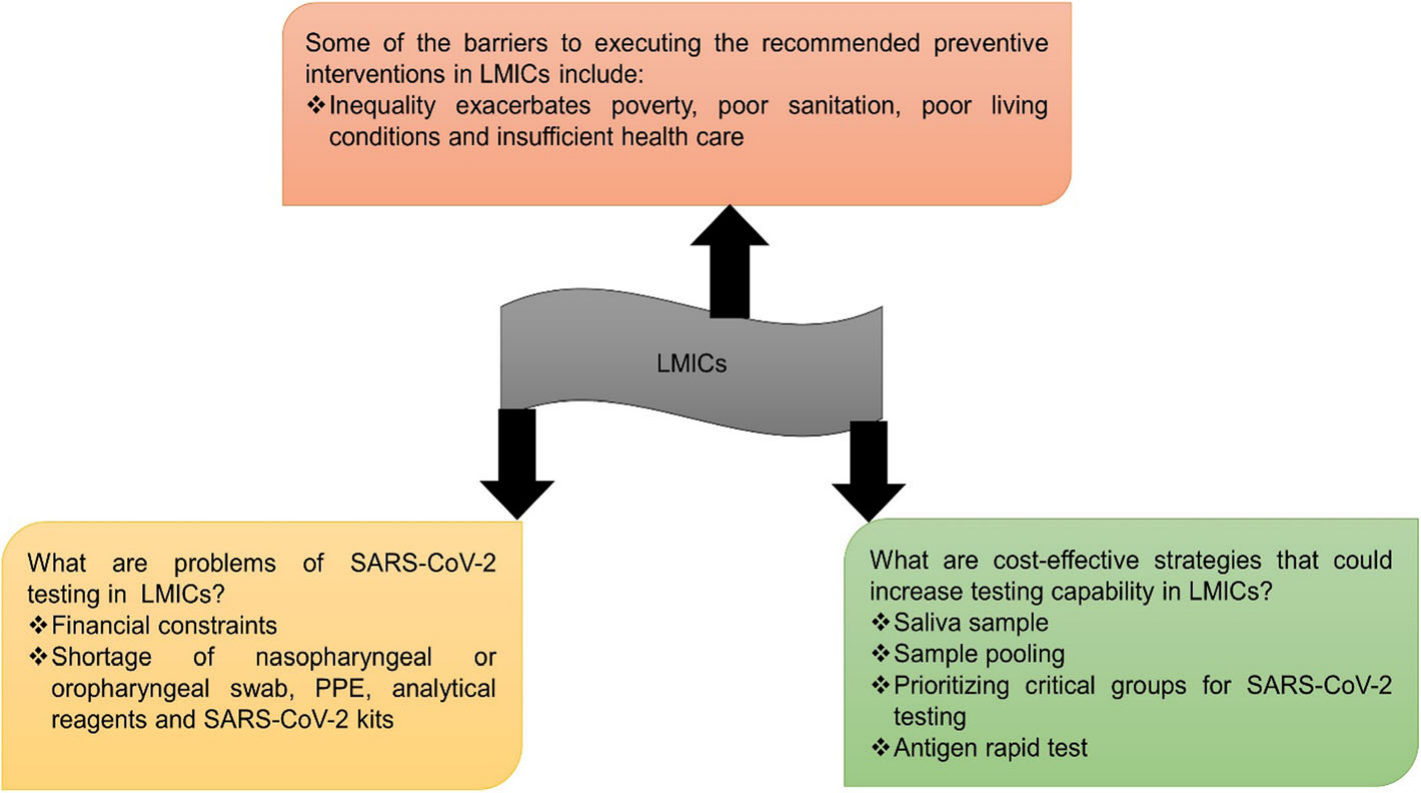
A summary of the major challenges that LMICs have when it comes to SARS-CoV-2 testing, as well as possible cost-effective strategies for increasing mass testing

In conclusion, as the number of reported cases rises, the pandemic’s long-term effect on individuals and populations in LMICs remains unknown. Moreover, the provision of a specific, effective vaccine to the people in LMICs is still a challenge. With an ongoing, unprecedented outbreak of SARS-CoV-2, the importance of laboratory detection of human coronavirus infections has been emphasized around the world in order to prevent the spread of the infection and properly treat those individuals who have a serious infection. However, due to weak health systems and poverty, LMICs are finding it difficult to manage the SARS-CoV-2 outbreak. This paper highlights the importance of developing alternative strategies for SARS-CoV-2 mass testing that are simple and cost-effective in a resource-constrained setting, and the summary is illustrated in Fig. 4.

For future research, the goal is to evaluate alternative methods that are simple and cheaper, with fast turn-around time and have a high throughput for a resource-constrained laboratory, so that they can be implemented to facilitate mass testing for SARS-CoV-2 infection in LMICs. Furthermore, such research will have a good impact on the development of a common pricing standard for SARS-CoV-2 kits in LMICs.

## Data Availability

All the data reported in this review was retrieved from the publicly available original sources.
